# Are We Sure We Fully Understand What an Infodemic Is? A Global Perspective on Infodemiological Problems

**DOI:** 10.2196/36510

**Published:** 2022-07-21

**Authors:** Alessandro Rovetta, Lucia Castaldo

**Affiliations:** 1 R&C Research Bovezzo (Brescia) Italy

**Keywords:** communication, conspiracy, COVID-19, education, fake news, infodemic, infodemiology, mass media, public health, risk perception, science

## Abstract

Infodemic is defined as an information epidemic that can lead to engaging in dangerous behavior. Although the most striking manifestations of the latter occurred on social media, some studies show that dismisinformation is significantly influenced by numerous additional factors, both web-based and offline. These include social context, age, education, personal knowledge and beliefs, mood, psychological defense mechanisms, media resonance, and how news and information are presented to the public. Moreover, various incorrect scientific practices related to disclosure, publication, and training can also fuel such a phenomenon. Therefore, in this opinion article, we seek to provide a comprehensive overview of the issues that need to be addressed to bridge the gap between science and the public and build resilience to the infodemic. In particular, we stress that the infodemic cannot be curbed by simply disproving every single false or misleading information since the belief system and the cultural or educational background are chief factors regarding the success of fake news. For this reason, we believe that the process of forming a critical sense should begin with children in schools (ie, when the mind is more receptive to new ways of learning). Furthermore, we also believe that themes such as scientific method and evidence should be at the heart of the university education of a future scientist. Indeed, both the public and scientists must be educated on the concepts of evidence and validity of sources, as well as learning how to dialogue appropriately with each other. Finally, we believe that the scientific publishing process could be greatly improved by paying reviewers for their work and by ceasing to pursue academic success at all costs.

## Infodemiology

Infodemiology was defined by Gunther Eysenbach “the science of distribution and determinants of information in an electronic medium, specifically the Internet, or in a population, with the ultimate aim to inform public health and public policy” [1,2]. The term was deliberately coined to recall epidemiology. Consequently, infodemic (ie, “epidemic” of information) represents the uncontrolled dissemination of information, including false or confusing information, during a disease outbreak [3-5]. To date, there is no univocal cataloging of the various types of infodemic information. For instance, disinformation is sometimes defined as the intersection between misinformation (eg, the creation of misleading content and false causal connections between phenomena) and malinformation (eg, leaks, harassment, and hate speech) [5]. On the contrary, Wang et al [4] argue that when the dissemination is voluntary and takes place for malicious purposes, we speak of disinformation; otherwise (ie, when it is unintentional and accidental) we speak of misinformation. Some authors enclose both meanings in the unique term “dismisinformation,” while others adopt the sometimes-criticized expression “fake news” [4,5]. Specifically, O’Hair et al [5] formally define dismisinformation as “any message or a set of messages that represent a meaning complex discrepant from or incompatible with a sender’s intent and/or a relatively informed or expert consensual evidentiary state.” In this regard, it is essential to point out that these denominations can include false news, polarized content, satire, misreporting, commentary, persuasive information, and citizen journalism [6]. In this paper, we will adopt the O’Hair et al [5] convention. Phenomena such as malinformation and conspiracy hypotheses will therefore be included in the concept of dis-misinformation. The importance of the infodemiological approach has always been known in the scientific community but was established definitively during the COVID-19 pandemic. In this regard, 132 states have signed an international document to guarantee their commitment to combat the COVID-19 infodemic as this has often resulted in damage of epidemiological and economic nature [3]. In this perspective paper, we address infodemiological issues, which, in our opinion, have been largely neglected by a significant fraction of the scientific community. Specifically, we will provide arguments to support the fact that the concept of dismisinformation is broader and more complex than it may seem at first glance.

## Effects of Communications on the Lay Neutral Public

Although infodemics cannot exist without dismisinformation, it is necessary to consider that even correct information (ie, based on facts and scientific evidence) contributes to its spread. Indeed, the juxtaposition of conflicting information only aggravates the negative influence on the lay public [7]. Such a contrast can arise and grow on two different levels: the dichotomy of reliable and unreliable news (Level 1, eg, scientific evidence versus dismisinformation) and the scientific debate (Level 2, eg, differing predictions based on preliminary data). Notable cases occurred during the COVID-19 pandemic. For example, fake news has emerged about the laboratory creation of SARS-CoV-2 as a virological weapon despite the scientific literature supporting the absence of voluntary manipulation [8]. Even more striking was the alleged correlation between 5G and the COVID-19 spread [9]. A well-trained scientist understands that such news is fake since the peer-reviewed scientific literature is, in the vast majority of cases, in agreement on the nonexistence of such phenomena. However, we must strive to put ourselves in the shoes of an inexperienced person. In particular, on average, a layperson does not have the basis for knowing the concept of “peer review” or “meta-analysis” and can distinguish the reliability of a source only up to a certain point. Let us take a concrete example. I turn on the television and hear about the side effects of COVID-19 vaccines [10]. Therefore, I start looking for details on the web, finding reassurance from my health organization [11]. Some time later, a friend of mine shares a video on Facebook where a doctor (or similar) talks about the severe damage of vaccines, denouncing an international conspiracy. Searching for information on the web, I find an article from the ByoBlu news channel, which confirms the doctor’s words; meanwhile, the vaccine debate becomes hot on talk shows [10]. Then, now in a panic, I ask for help from my general practitioner, who turns out to be a convinced “anti-vaxxer” [12,13]. Hence, I decide not to vaccinate myself, and I advise my family and friends against vaccines. Unfortunately, this is a realistic scenario, as evidenced by the sources mentioned. Furthermore, the above example makes the distinction of Level 1 from Level 2 extremely relevant and subtle. In fact, who is to blame for this irrational reaction? To have a reasoned answer, we need to analyze what happened. First of all, we must consider that the influence of mass media on the population is still extremely high today [14]. Secondly, the conflicting reports, even coming from doctors and scientists, create confusion and diminish trust in the authorities [3]. Rationality gives way to anxiety and fear, increasing the likelihood of assuming harmful behaviors, in this case, not being vaccinated against COVID-19 [3,15]. Indeed, vaccine hesitancy is fueled by the constant discussion on their side effects due to the cognitive distortion of risk perception [10,16]. Such a distortion is reinforced by the fact that sensationalistic headlines can create a bias in reading or listening to the news, and the emotional impact on risk perception is, on average, much higher than that of a logical argument [17,18]. Therefore, the explanations of these phenomena are to be divided between inappropriate communication and personal unpreparedness. While the reasons for writing shocking titles and reporting news with unnecessary emphasis are related to acquiring more audience and clickbaiting [19], the personal inability to process information rationally does not derive simply or solely from one’s willingness not to. In this regard, the World Health Organization (WHO) has firmly stated that we must build resilience to infodemics [3]. Currently, we believe that national school programs are generally inadequate to form the critical and analytical sense necessary to weigh the risk perception based on the available scientific evidence. Specifically, we believe people are not guided and educated on how to judge the trustworthiness of a source. Moreover, even teachers and professors are not prepared to deal with such a vast and complicated topic. Therefore, we believe that the first fundamental step in addressing the future infodemic is creating a school program suitable for the formation of resilience to dismisinformation (Point 1). As a matter of fact, changing a psychological or behavioral attitude beyond a specific age group becomes difficult [20-23], which requires acting on the malleable minds of young people to help them become whole and independent people. Similar conclusions on the importance of health education for children and young people were reached by MacDonald et al [24]. These strategies must be added to what is already being carried out to combat the infodemic [3].

## The Plague of Conspiracy Hypotheses

Conspiracy thinking originates from questionings of various kinds, including epistemic, existential, and social [25]. There is evidence that these attitudes are the aberration of mechanisms useful for the human race’s survival, such as pattern recognition, agency detection, threat management, alliance, and dangerous coalitions detection [26]. For instance, the rejection of medical science is caused by complex and unconscious phenomena, including but not limited to illusory truth phenomenon (repeated exposure to falsehood can prime us to accept it implicitly), the availability heuristic (we afford more weight to more readily recalled information, even when this might be misleading), and the fallacy of anecdotal vividness (we tend to react more viscerally to emotive claims than more sober-headed analysis) [27]. Moreover, the Dunning-Kruger effect, which states that incompetents overestimate their knowledge on a particular topic, feeds the conspirationism [28]. This makes communication with these people very difficult as they are excessively prejudicial and do not have the technical means to understand why they are wrong. The press and media coverage of fake news does not help, as confirmatory biases drive conspirationists [10]. In these cases, the implementation of infoveillance and content remotion systems such as those adopted by social platforms could be the only way to limit, at least temporarily, the infodemic on the web [14]. Nonetheless, as discussed in the previous section, conspiracy hypotheses also come from people expected to demonstrate high competence (eg, doctors and scientists). The most striking case is that of Nobel Laureate Luc Montagnier, a staunch supporter of the no-vax movement who has fostered the hypothesis that SARS-CoV-2 was born from a voluntary manipulation of HIV [29,30]. Such incidents have been far from isolated, as evidenced by many professors, doctors, and nurses demonstrating skepticism and unfounded views on vaccines [12,13,31]. In particular, Paris et al [31] highlighted the devastating impact of media communication about vaccine side effects on the class of health care workers. In this regard, it is crucial to keep in mind that it is often the social context (eg, an ingrained belief system) that makes conspiracy theorists appealing to the public [32]. At the same time, Heyerdahl et al [33] showed that fear of peer judgment prompted many health care professionals not to express their doubts about COVID-19 vaccination. This evidence exhibits that not even these people’s scientific training has been sufficient to manage the infodemic. Furthermore, conspiracy and dread often mix in a murky sea that makes them almost indistinguishable. Therefore, we believe that scientific training should focus more on adopting the scientific method and analyzing sources’ reliability (Point 2). Specifically, a science graduate should master the concept of “degrees of evidence” (eg, original article vs meta-analysis) and the credibility of a source (eg, nondeposited preprint vs peer-reviewed article). On this point, we also believe it is essential that the principle of authority be minimized; the conviction of being an expert in the sector must not induce us to think that we can ignore the most recent scientific evidence. A scientist is a real scientist only if they are constantly willing to question what they know based on the most updated literature.

## Problems in Scientific Communication

Beyond the glaring errors of the press and conspiratorial characters, including scientists, we must ask ourselves the following: has the communication from the scientific community been adequate? On January 14, 2020, the WHO wrote on the official Twitter account “Preliminary investigations conducted by the Chinese authorities have found no clear evidence of human-to-human transmission of the novel #coronavirus (2019-nCoV) […]” [34]. This statement means that, at the moment, the scientific community does not know if the novel coronavirus 2019-nCoV can be transmitted from human to human. The subsequent day, Maria Van Kerkhove stated in a press conference “From the information that we have it is possible that there is limited human-to-human transmission, potentially among families, but it is very clear right now that we have no sustained human-to-human transmission” [35,36]. The first part of the statement is very cautious, as it is weighted on expressions such as “From the information that we have” and “it is possible.” On the contrary, the second sentence alludes to an implausible possibility, that is, that there is clear evidence to affirm that the virus is not transmitted easily from person to person. In fact, this affirmation was soon denied not only by robust evidence of transmission from symptomatic infected patients but also from presymptomatic and asymptomatic patients [37-39]. Information channels with a large audience shared this news adding further inaccuracies. For example, Reuters published 2 articles with the same title 5 minutes apart. In the first, the opening sentence used the verb “has [limited transmission]” [40], while in the second (the US version), the wording “may have” was adopted [41]. In summary, we have confusing, slightly inaccurate, and covertly contradictory information presented to an inexperienced audience. Even worse is the media debate that arose before the pandemic outbreak in Europe. For example, in Italy, scientists provided diametrically opposed opinions on the severity of COVID-19, breaking public opinion in half [10,14]. This contributed to the emergence of serious protests when the implementation of lockdowns was requested, which proved to be a fundamental tool in cutting down the number of cases and saving millions of lives [42]. In such delicate times, words are just boulders and must be chosen carefully. Indeed, communication errors of this type can provide material for conspiracy hypotheses and confuse the public trying to orient themselves within a new dramatic and unusual situation. Beyond the mistakes of the press for which they are not responsible, scientists have a moral obligation to predict the public reaction to certain circumstances as they possess an additional intellectual tool, that is, the scientific method. When it is necessary to communicate sensitive information, it would be advisable to write the texts or at least the essential passages of the latter to ensure the greatest possible clarity and precision. Furthermore, when consulted, doctors and professors must behave scientifically, that is, base their claims on the degree of evidence available in the literature (Point 3). In this respect, we would like to share the experience and thoughts of one of the authors.

On several occasions during COVID-19, I have had discussions with some scientists who were well-known faces of the web and Italian television. The latter’s nature concerned an aspect that I consider of absolute communicative importance: the scientific validity of public statements. For example, many argued COVID-19–related opinions on their Facebook public profiles based on a single preprint without specifying that those results were not peer reviewed nor confirmed by other literature. One of them even replied that this clarification was unnecessary since he was a peer reviewer, as if a single reviewer could replace the entire peer-review process that includes two or more reviewers—depending on the topic’s relevance—and an editor’s final judgment. What surprises me is the arrogance of people who think they can be above the scientific method and community because they have an academic title or role. All this while we have had direct proof that even Nobel laureates can assert dangerous unscientific nonsense. What lesson is being taught to the public by acting this way? Such an excessive usage of the principle of authority distances us from facts and credible communication and urges the public to give importance to the individual rather than the available evidence.

AR

## Current Challenges in Scientific Publishing and Disclosure

Returning to the previous section, we ask ourselves, “what is the reason that prompts scientists to share comments on preprints or other forms of nonpeer-reviewed literature?” During the COVID-19 pandemic, rapid and timely interventions were significant public health challenges [43]. Since COVID-19 depends on many factors and comorbidities and the variants of concern can substantially change its behavior [44-46], having reliable updated data in short times is an essential aspect of containing outbreaks. Unfortunately, the peer review and publication processes are inadequately slow to face a health crisis properly. Huisman et al [47] found that only 13%-16% of papers covering medicine, natural sciences, and public health were accepted within 1 month, and the average acceptance time ranged from 12 to 14 weeks [47]. In our experience as reviewers and authors for over 50 scientific journals during the COVID-19 pandemic, we encountered very long publication times, even for articles with a high scientific impact (eg, side effects of COVID-19 vaccines). Therefore, we understand and agree on the need to comment on preliminary findings as long as it is openly stated that these results are uncertain, and the meaning of “preprint” is clearly explained to the public. Furthermore, national and supranational agencies such as the Centers for Disease Control and Prevention (CDC), the European Medicines Agency (EMA), Food and Drug Administration, and the WHO are constant sources of up-to-date, internally reviewed data and can be consulted to obtain credible and calibrated news on the available evidence. Finally, we firmly believe that peer reviews should give absolute priority to Methods and Results over other sections, and journals should report methodological acceptance of the manuscript publicly. By doing so, researchers in the field—who are unlikely to need the Introduction and Discussion sections to understand and contextualize the paper—could receive new data more promptly. We must remember that science, especially medicine, saves lives. Delaying the publication of a manuscript for aesthetics, layout, or sections not essential for its reproducibility is just an unjustifiable academic caprice. Unfortunately, as things stand, a peer reviewer is required to report these aspects, and journals should be the ones to change their editorial policy.

Alongside this, the scientific community has to deal with internal situations. The first we want to discuss is predatory publication. Predatory journals offer rapid publication times at meager costs, making them very attractive to independent researchers who do not have funds available. However, these apparent benefits arise from poor or bogus peer-review processes [48]. Hence, the researchers identified various strategies to combat this phenomenon, including creating lists of predatory scientific journals and publishers and bibliographic abstracting and indexing [49,50]. Nonetheless, the inclusion criteria in predatory blacklists have always been the subject of criticisms and controversies, and predatory publications have managed to slip into prestigious repositories such as PubMed [51,52]. Therefore, there is no foolproof way but only general indications to recognize and avoid predatory publishers. The predatory phenomenon could be provoked and sustained by the success of the open access publishing method or the excessive editorial costs of renowned journals [53,54]. Nevertheless, even the rush to publish their results can push an author to choose alternative routes to standard publication. The second issue we want to discuss concerns the scientific role of peer review. Specifically, peer review is a fundamental procedure to ensure the accuracy of manuscripts published in scientific journals [55]. The independent judgment of 2 or more expert scientists not involved in the study examined and without conflicts of interest is a first step to skim the literature from gross errors. At the same time, a single article with new results is always a low degree of evidence until other studies confirm its findings. In fact, peer review still presents several flaws, including a possible low agreement between the referees [56-58]. In confirmation of this, it is not surprising to find numerous withdrawn articles [59]. Therefore, peer review is not and cannot be the final judgment on a scientific paper. Based on these premises, Adler [60] has proposed a quick review method that also includes a postpublication review. Yet this approach was not unanimously accepted by the scientific community. Checco et al [61] recently proposed semiautomated artificial intelligence peer review systems capable of assisting the reviewer and improving the review quality, but the authors conclude that there are still concerns to be settled. The third issue we want to highlight regards the conflict of interest of journal publications and the unpaid contribution of peer reviewers. Peer review is a time-consuming and challenging process. Some researchers believe paying reviewers could facilitate shoddy reviews with the sole scope of getting the money reward [62]. In this regard, the authors of this paper—and many other scientists [62]—consider this statement to be simply false for various reasons, including the following: First, reviewers are selected by editors responsible for judging their reliability; second, the same criticism can also be raised for unpaid reviews as a referee could carry out hasty reports to obtain easy certification for personal prestige and curriculum. Third, paid reviewers would transform peer review into a real job with merit-based selection. In this regard, getting paid to conduct a review could increase the competition for top-notch reviews. Moreover, we believe that it would be far more rewarding and fairer for reviewers to have their work generate income. In our experience, a concrete example is that of JMIR Publications; if the editor evaluates the review of a paper to be of sufficient quality, the reviewer can earn up to 100 dollars to spend to publish in one of their journals [63]. Even if it is something very distant from a real waged job, this can be a first step in proving that the model is perfectly sustainable. Beyond that, we consider the association “paid reviewer-compromised review” hypocritical within the current editorial context. Indeed, the paid publication itself is basically a conflict of interest since journals only receive money if the articles are published. For instance, the standard policy of publishers asserts that a paper retraction does not involve the return of the article process charge to the authors (ie, editors may be motivated to publish regardless of the quality of the manuscript) [64]. Notwithstanding that, forcing the public to pay to read is not an acceptable solution if the goal is to keep the scientific community and the population updated on the latest evidence. The question, therefore, is “what to do then?” The open-access model is now widespread, and there is sufficient evidence that paying reviewers does not compromise the scientific quality of manuscripts. Since science continues to work, we believe that this is the right way to follow, with the awareness that the final judgment comes from the scientific community and not from the peer review. The fourth critical aspect concerns the search for scientific prestige. Many authors are convinced that metrics such as impact factor and number of citations are quality indices, while a vast scientific literature demonstrates that such indices are unreliable and can even be misleading [65]. Two of the main reasons are that impact factor is primarily influenced by outliers (ie, very few papers with very high citations), and the citations could reflect more the media success of an article than its quality (ie, more in-depth articles may not be known). In addition, it is necessary to consider why a paper was cited (eg, many citations concern introductory outline aspects). Just as the first duty of peer reviewers is not to be influenced by the prestige of the authors, the first duty of researchers is not to be affected by the reputation of journals when asked to evaluate the scientific content of a paper. As proof of this, numerous preprints have received thousands of positive citations inherent to the methodology adopted [66]. But the obsessive pursuit of prestige can also plague publishers. For instance, editorial reluctance to publish negative or null results can strongly bias literature [67,68]. Regarding all this, we support the healthy desire to be deemed great scientists and receive well-deserved gratifications as long as these do not lead to scientific discrimination and bias. However, considering the above evidence, part of the scientific community seems more interested in self-achievements rather than facts, and we need to change this. The fifth and final point concerns the secrecy of peer reviews. On January 16, 2020, Thijs Kuiken was contacted by “The Lancet” journal to review a paper within 48 hours [69]. The research showed strong evidence that SARS-CoV-2 was transmissible between humans, but the reviewer was expected to keep silent to protect the integrity of the evaluation and the authors’ and journal’s rights. Nonetheless, Thijs Kuiken faced this ethical dilemma with courage and conscientiousness and found a way to communicate the data to the WHO quickly. This episode testifies that the omission of essential public health information must be viewed as full-fledged disinformation. We stress that rapid medical evidence can save human lives and must be prioritized above everything else, especially during health crises. Failure to immediately communicate novel results to the authorities worldwide is a serious wrongdoing, and we must avoid it in the proximal future. Therefore, a standard procedure to deal with these exceptional situations must be designed and implemented as soon as possible.

## Degrees of Reliability and Final Recommendations

The concepts of degree (or level) of evidence have long been addressed in the literature and differ by health discipline [70]. Various changes have been made over time, attempting to improve the standard classifications [71]. Despite this, as shown in the previous sections, this element is culpably left out in most public statements. Further critical issues arise when presenting sensitive information to the public; indeed, it is not just a matter of communicating the degree of evidence (eg, original article vs meta-analysis) but also its credibility (eg, publication in a predatory journal vs publication in a legitimate journal). Therefore, the public should be educated on what we have termed “degree of reliability,” that is, a scale that considers both the level of evidence and the credibility of scientific works. Doing so would limit the damage of conflicting information since not all pieces of information would have the same reliability anymore. Therefore, we recommend that health agencies establish a standard classification of degrees of reliability that can be adopted internationally for each discipline by each practitioner. In the meantime, all medical professionals should adopt and specify the scale they deem most appropriate before expressing public statements on sensitive topics. As regards other types of information (eg, facts and fake news checks), we propose the following 6 hierarchy of degrees of reliability, starting from the letter “F” all the way to the letter “A.”

1. Class F: regardless of their authority, personal opinions must be ranked with the lowest degree of evidence. This is necessary to ensure consistency in class attribution. In particular, the authority principle must be minimized to prevent unjustified claims (eg, Montagnier) from sowing doubt, fear, and false information. Therefore, all conclusions that have not been moderated or peer reviewed fall into this category.
2. Class E: preprint moderation avoids the circulation of very serious fake news in the scientific world, but it is not comparable to a peer-review process. Indeed, a large number of preprints fueling conspiracy theories and unjustified assumptions have been unearthed [72]. Therefore, although the probability of finding infodemics is lower than the previous category, moderated preprints do not represent sufficient evidence to make scientific and especially medical claims. Besides, it is essential to consider that screening criteria are not uniform between the various preprint platforms; for instance, medRxiv and bioRxiv repositories operate stricter selection criteria about COVID-19 than other databases [73]. Hence, it is also necessary to consider this aspect when evaluating the classification level.
3. Class D: this category encompasses academic journals not yet indexed in recognized databases (eg, due to their novelty) and not listed among predatory journals (eg, Beall’s List). The degree of evidence is sufficient to expose assessments to the public, provided that it is specified that these are premature analyzes. Furthermore, inclusion in this class implies that an extensive literature search has been implemented, ascertaining the absence of known opposite results. If the number of articles reporting contrary findings is comparable, then the scientist is required to express a personal judgment (eg, comparing the validity of the 2 studies on their scope); in this case, the quality of the evidence is class E. If the number of articles supporting contrary results is significantly greater, then the scientist is required to make a personal judgment and present the quality of the evidence as class F.
4. Class C: the criteria of point D apply, but the probability of disseminating infodemic material further decreases thanks to bibliographic indexing.
5. Class B: the criteria of point C apply, but there must be at least 3 agreeing articles. Evidence proposed by recognized health agencies (eg, EMA, CDC, and WHO) can fall into this category.
6. Class A: the criteria in point B apply, but the articles must be systematic meta-analyses or reviews. Evidence proposed by recognized health agencies (eg, EMA, CDC, and WHO) can fall into this category.

We stress that this scale is indicative and needs to be further elaborated by the scientific community, including all the particular cases that have escaped us, for improving the basic setting. However, we believe it can serve as a general guideline, showing a possible way forward to limit the infodemic drastically. Indeed, the simple fact of specifying the sources and the type of evidence proposed can give the public an idea of the news’ relevance and weight (Table 1).

A colored version of Table 1, useful for dissemination, is presented as Multimedia Appendix 1.

**Table 1:** Scientific communication quality classes.

Class quality	Color-related name	Complete name	Evaluation description
F	Red	Opinion (very poor reliability)	Based on raw data, personal experience, or preprint not deposited on accredited preprint repositories.
E	Orange	Indexed novel preprint (poor reliability)	Based on a new preprint deposited on one or more accredited preprint repositories.
D	Yellow	Unindexed new article (uncertain reliability)	Based on a new article not deposited on accredited article repositories. Known predatory journals are excluded.
C	Green	Indexed article (fair reliability)	Based on 1 or 2 articles deposited on one or more accredited article repositories or affirmed antihoax nongovernment websites.
B	White	Evidence (good reliability)	Based on 3 or more^a^ articles or highly and properly cited preprints deposited on accredited article repositories or accredited gray literature.
A	Azure	Confirmed evidence (very good reliability)	Evaluation based on systematic review articles or meta-analyses deposited on accredited article repositories or accredited gray literature.

^a^This number may change depending on the importance of the evidence (eg, much more evidence may be required on drug-related information).

## Appendix

Multimedia Appendix 1 Scientific communication quality classes. The marked line highlights the sufficiency threshold. (a) This number may change depending on the importance of the evidence (eg, much more evidence may be required on drug-related information).

## Abbreviations

CDC: Centers for Disease Control and Prevention
EMA: European Medicines Agency
WHO: World Health Organization

## References

[1] Eysenbach G (2009). Infodemiology and infoveillance: framework for an emerging set of public health informatics methods to analyze search, communication and publication behavior on the Internet. J Med Internet Res.

[2] Mackey T, Baur C, Eysenbach G (2022). Advancing Infodemiology in a Digital Intensive Era. JMIR Infodemiology.

[3] Infodemic. World Health Organization.

[4] Wang Y, McKee M, Torbica A, Stuckler D (2019). Systematic Literature Review on the Spread of Health-related Misinformation on Social Media. Soc Sci Med.

[5] O’Hair HD, O’Hair MJ (2021). Communicating Science in Times of Crisis: COVID-19 Pandemic.

[6] Molina MD, Sundar SS, Le T, Lee D (2019). “Fake News” Is Not Simply False Information: A Concept Explication and Taxonomy of Online Content. American Behavioral Scientist.

[7] Nagler RH, Yzer MC, Rothman AJ (2019). Effects of Media Exposure to Conflicting Information About Mammography: Results From a Population-based Survey Experiment. Ann Behav Med.

[8] Maxmen A, Mallapaty S (2021). The COVID lab-leak hypothesis: what scientists do and don't know. Nature.

[9] Ahmed W, Vidal-Alaball J, Downing J, López Seguí F (2020). COVID-19 and the 5G Conspiracy Theory: Social Network Analysis of Twitter Data. J Med Internet Res.

[10] Rovetta A (2021). The Impact of COVID-19 on Conspiracy Hypotheses and Risk Perception in Italy: Infodemiological Survey Study Using Google Trends. JMIR Infodemiology.

[11] Vaccini anti Covid-19. Ministero della Salute.

[12] Le Marechal M, Fressard L, Agrinier N, Verger P, Pulcini C (2018). General practitioners' perceptions of vaccination controversies: a French nationwide cross-sectional study. Clin Microbiol Infect.

[13] Aggressione di medici No-Vax durante assemblea Ordine di Roma. Quotidiano Sanità.

[14] Rovetta A, Castaldo L (2021). Influence of Mass Media on Italian Web Users During the COVID-19 Pandemic: Infodemiological Analysis. JMIRx Med.

[15] Troiano G, Nardi A (2021). Vaccine hesitancy in the era of COVID-19. Public Health.

[16] Tversky A, Kahneman D (1973). Availability: A heuristic for judging frequency and probability. Cognitive Psychology.

[17] Ecker UKH, Lewandowsky S, Chang EP, Pillai R (2014). The effects of subtle misinformation in news headlines. J Exp Psychol Appl.

[18] Megías A, Cándido A, Maldonado A, Catena A (2018). Neural correlates of risk perception as a function of risk level: An approach to the study of risk through a daily life task. Neuropsychologia.

[19] Bolton DM, Yaxley J (2017). Fake news and clickbait - natural enemies of evidence-based medicine. BJU Int.

[20] Xie B, Watkins I, Golbeck J, Huang M (2012). Understanding and Changing Older Adults' Perceptions and Learning of Social Media. Educ Gerontol.

[21] Vaportzis E, Clausen MG, Gow AJ (2017). Older Adults Perceptions of Technology and Barriers to Interacting with Tablet Computers: A Focus Group Study. Front Psychol.

[22] Cookman CA (1996). Older people and attachment to things, places, pets, and ideas. Image J Nurs Sch.

[23] Matamales M, Skrbis Z, Hatch RJ, Balleine BW, Götz J, Bertran-Gonzalez J (2016). Aging-Related Dysfunction of Striatal Cholinergic Interneurons Produces Conflict in Action Selection. Neuron.

[24] MacDonald NE, Dubé E (2018). Addressing vaccine hesitancy in immunization programs, clinics and practices. Paediatr Child Health.

[25] Douglas KM, Sutton RM, Cichocka A (2017). The Psychology of Conspiracy Theories. Curr Dir Psychol Sci.

[26] van Prooijen J, van Vugt M (2018). Conspiracy Theories: Evolved Functions and Psychological Mechanisms. Perspect Psychol Sci.

[27] Grimes DR (2021). Medical disinformation and the unviable nature of COVID-19 conspiracy theories. PLoS One.

[28] Gonçalves-Sá J (2020). In the fight against the new coronavirus outbreak, we must also struggle with human bias. Nat Med.

[29] Arif N, Al-Jefri M, Bizzi IH, Perano GB, Goldman M, Haq I, Chua KL, Mengozzi M, Neunez M, Smith H, Ghezzi P (2018). Fake News or Weak Science? Visibility and Characterization of Antivaccine Webpages Returned by Google in Different Languages and Countries. Front. Immunol.

[30] Sallard E, Halloy J, Casane D, Decroly E, van Helden J (2021). Tracing the origins of SARS-COV-2 in coronavirus phylogenies: a review. Environ Chem Lett.

[31] Paris C, Bénézit F, Geslin M, Polard E, Baldeyrou M, Turmel V, Tadié É, Garlantezec R, Tattevin P (2021). COVID-19 vaccine hesitancy among healthcare workers. Infect Dis Now.

[32] Drążkiewicz E (2022). Study conspiracy theories with compassion. Nature.

[33] Heyerdahl LW, Dielen S, Nguyen T, Van Riet C, Kattumana T, Simas C, Vandaele N, Vandamme A, Vandermeulen C, Giles-Vernick T, Larson H, Grietens KP, Gryseels C (2022). Doubt at the core: Unspoken vaccine hesitancy among healthcare workers. Lancet Reg Health Eur.

[34] Preliminary investigations conducted by the Chinese authorities have found no clear evidence of human-to-human transmission of the novel #coronavirus (2019-nCoV) identified in #Wuhan, #China. World Health Organization.

[35] (2020). Wuhan virus has limited human-to-human transmission but could spread wider: WHO. The Straits Times.

[36] (2020). 'Possible' there was limited human-to-human transmission of new coronavirus in China, WHO says. Canadian Broadcasting Corporation.

[37] Gandhi M, Yokoe DS, Havlir DV (2020). Asymptomatic Transmission, the Achilles' Heel of Current Strategies to Control Covid-19. N Engl J Med.

[38] Sah P, Fitzpatrick MC, Zimmer CF, Abdollahi E, Juden-Kelly L, Moghadas SM, Singer BH, Galvani AP (2021). Asymptomatic SARS-CoV-2 infection: A systematic review and meta-analysis. Proc Natl Acad Sci U S A.

[39] Cevik M, Kuppalli K, Kindrachuk J, Peiris M (2020). Virology, transmission, and pathogenesis of SARS-CoV-2. BMJ.

[40] (2020). WHO says new China virus could spread, it's warning all hospitals. Reuters.

[41] Nebehay S (2020). WHO says new China virus could spread, it's warning all hospitals. Reuters.

[42] Talic S, Shah S, Wild H, Gasevic D, Maharaj A, Ademi Z, Li X, Xu W, Mesa-Eguiagaray I, Rostron J, Theodoratou E, Zhang X, Motee A, Liew D, Ilic D (2021). Effectiveness of public health measures in reducing the incidence of covid-19, SARS-CoV-2 transmission, and covid-19 mortality: systematic review and meta-analysis. BMJ.

[43] Rypdal K, Bianchi FM, Rypdal M (2020). Intervention Fatigue is the Primary Cause of Strong Secondary Waves in the COVID-19 Pandemic. Int J Environ Res Public Health.

[44] Pluchino A, Biondo AE, Giuffrida N, Inturri G, Latora V, Le Moli R, Rapisarda A, Russo G, Zappalà C (2021). A novel methodology for epidemic risk assessment of COVID-19 outbreak. Sci Rep.

[45] (2022). People with Certain Medical Conditions. Centers for Disease Control and Prevention (CDC).

[46] Hadj Hassine I (2021). Covid-19 vaccines and variants of concern: A review. Rev Med Virol.

[47] Huisman J, Smits J (2017). Duration and quality of the peer review process: the author's perspective. Scientometrics.

[48] Elmore SA, Weston EH (2020). Predatory Journals: What They Are and How to Avoid Them. Toxicol Pathol.

[49] Strinzel M, Severin A, Milzow K, Egger M (2019). Blacklists and Whitelists To Tackle Predatory Publishing: a Cross-Sectional Comparison and Thematic Analysis. mBio.

[50] Dhammi IK, Haq RU (2016). What is indexing. Indian J Orthop.

[51] Vakil C (2019). Predatory journals: Authors and readers beware. Can Fam Physician.

[52] Manca A, Moher D, Cugusi L, Dvir Z, Deriu F (2018). How predatory journals leak into PubMed. CMAJ.

[53] Shen C, Björk B (2015). 'Predatory' open access: a longitudinal study of article volumes and market characteristics. BMC Med.

[54] Harvard University says it can't afford journal publishers' prices. The Guardian.

[55] Ali PA, Watson R (2016). Peer review and the publication process. Nurs Open.

[56] Hope AA, Munro CL (2019). Criticism and Judgment: A Critical Look at Scientific Peer Review. Am J Crit Care.

[57] Pier EL, Brauer M, Filut A, Kaatz A, Raclaw J, Nathan MJ, Ford CE, Carnes M (2018). Low agreement among reviewers evaluating the same NIH grant applications. Proc Natl Acad Sci U S A.

[58] Oxman AD, Guyatt GH, Singer J, Goldsmith CH, Hutchison BG, Milner RA, Streiner DL (1991). Agreement among reviewers of review articles. J Clin Epidemiol.

[59] Anderson C, Nugent K, Peterson C (2021). Academic Journal Retractions and the COVID-19 Pandemic. J Prim Care Community Health.

[60] Adler JR (2012). A new age of peer reviewed scientific journals. Surg Neurol Int.

[61] Checco A, Bracciale L, Loreti P, Pinfield S, Bianchi G (2021). AI-assisted peer review. Humanit Soc Sci Commun.

[62] Brainard J (2021). The $450 question: Should journals pay peer reviewers?. Science.

[63] Karma Credits - What are they and how to collect them?. JMIR Publications.

[64] Wadman M (2021). Scientists quit journal board, protesting ‘grossly irresponsible’ study claiming COVID-19 vaccines kill. Science.

[65] Rovetta A (2021). it is ridiculous and shameful that part of the scientific community adopts the impact factor as an index of the quality of an academic journal or, worse, of the articles published in it. Facebook.

[66] Chen T, Guestrin C (2016). XGBoost: A Scalable Tree Boosting System. ACM Digital Library.

[67] Matosin N, Frank E, Engel M, Lum JS, Newell KA (2014). Negativity towards negative results: a discussion of the disconnect between scientific worth and scientific culture. Dis Model Mech.

[68] Bespalov A, Steckler T, Skolnick P (2019). Be positive about negatives-recommendations for the publication of negative (or null) results. Eur Neuropsychopharmacol.

[69] De Vrieze J (2021). An unpublished COVID-19 paper alarmed this scientist—but he had to keep silent. Science.

[70] van Dijk WB, Grobbee DE, de Vries MC, Groenwold RHH, van der Graaf R, Schuit E (2019). A systematic breakdown of the levels of evidence supporting the European Society of Cardiology guidelines. Eur J Prev Cardiol.

[71] Burns PB, Rohrich RJ, Chung KC (2011). The levels of evidence and their role in evidence-based medicine. Plast Reconstr Surg.

[72] Koerber A (2020). Is It Fake News or Is It Open Science? Science Communication in the COVID-19 Pandemic. Journal of Business and Technical Communication.

[73] Kwon D (2020). How swamped preprint servers are blocking bad coronavirus research. Nature.

