# Supplementary material for: Are We Sure We Fully Understand What an Infodemic Is? A Global Perspective on Infodemiological Problems
Source: JMIRx Med. 2022 Jul 21;3(3):e36510. doi: 10.2196/36510 (PMC9642843; doi:10.2196/36510)
Supplement: Multimedia Appendix 1 [file xmed_v3i3e36510_app1.pdf]

Class Quality	Color-related name	Complete name	Evaluation description
F	Red	Opinion (very poor validity)	Based on raw data, personal experience, or pre-print not deposited on *accredited preprint repositories*.
E	Orange	Indexed novel preprint (poor validity)	Based on a new preprint deposited on one or more accredited preprint repositories.
D	Yellow	Unindexed new article (uncertain validity)	Based on a new article not deposited on *accredited article repositories*. Known predatory journals are excluded.
C	Green	Indexed article (fair validity)	Based on 1 or 2articles deposited on one or more accredited article repositories or affirmed anti-hoax non-government websites.
B	White	Evidence (good validity)	Based on 3 or more[a] articles or highly and properly cited preprints deposited on accredited article repositories or accredited gray literature.
A	Azure	Confirmed Evidence (very good validity)	Evaluation based on systematic review articles or meta-analyses deposited on accredited article repositories or accredited gray literature.

Table 1. Scientific communication quality classes. The marked line highlights the sufficiency threshold. [a]This number may change depending on the importance of the evidence (e.g., much more evidence may be required on drug-related information).

F. Regardless of their authority, personal opinions must be ranked with the lowest degree of evidence. This is necessary to ensure consistency in class attribution. In particular, the authority principle must be minimized to prevent unjustified claims (e.g., Montagnier) from sowing doubt, fear, and false information. Therefore, all conclusions that have not been moderated or peer-reviewed fall into this category.

E. Preprints moderation avoids the circulation of very serious fake news in the scientific world, but it is not comparable to a peer-review process. Indeed, a large number of preprints fueling conspiracy theories and unjustified assumptions have been unearthed [68]. Therefore, although the probability of finding infodemics is lower than the previous category, moderated preprints do not represent sufficient evidence to make scientific and especially medical claims.

D. This category encompasses academic journals not yet indexed in recognized databases (e.g., due to their novelty) and not listed among predatory journals (e.g., Beall's List). The degree of evidence is sufficient to expose assessments to the public, provided that it is specified that these are premature analyzes. Furthermore, inclusion in this class implies that an extensive literature search has been done, ascertaining the absence of known opposite results. If the

number of articles reporting contrary findings is comparable, then the scientist is required to express a personal judgment (e.g., comparing the validity of the two studies on their scope); in this case, the quality of the evidence is class E. If the number of articles supporting contrary results is significantly greater, then the scientist is required to make a personal judgment and present the quality of the evidence as class F.

C. The criteria of point D apply, but the probability of disseminating infodemic material further decreases thanks to bibliographic indexing.

B. The criteria of point C apply, but there must be at least three agreeing articles. Evidence proposed by recognized health agencies (e.g., EMA, CDC, WHO) can fall into this category.

A. The criteria in point B apply, but the articles must be systematic meta-analyzes and/or reviews. Evidence proposed by recognized health agencies (e.g., EMA, CDC, WHO) can fall into this category.

We stress that this scale is indicative and needs to be further elaborated by the scientific community, including all the particular cases that have escaped us, for improving the basic setting. However, we believe it can serve as a general guideline, showing a possible way forward to limit the infodemic drastically. Indeed, the simple fact of specifying the sources and the type of evidence proposed can give the public an idea of the news' relevance and weight
